# Inherited and *de novo* germline TP53 mutations in adult-onset sarcoma

**DOI:** 10.1186/1897-4287-10-S2-A3

**Published:** 2012-04-12

**Authors:** DM Thomas, ML Ballinger

**Affiliations:** 1 Research Division, Peter MacCallum Cancer Centre, East Melbourne, VIC, Australia

## 

The International Sarcoma Kindred Study (ISKS) was initiated in 2009 to investigate the prevalence and nature of heritable risk in sarcoma populations. Sarcomas affect a younger population than most cancers, and are associated with some of the most striking known familial cancer syndromes. For example, the Li-Fraumeni syndrome (LFS) is a devastating heritable condition, conferring a 90% lifetime risk of cancer and a 50% risk of invasive cancer by age 30. LFS is predominantly due to germline mutations in TP53 and CHEK2. Sarcomas collectively comprise the most frequent cancer seen in LFS. The ISKS is recruiting all adult patients diagnosed with sarcoma in Australia, regardless of family history. Recently, ISKS opened sites in New Zealand and India, and plans to open in France later this year.

At June 2011, the ISKS has recruited over 450 adult sarcoma probands and their families. The average age of onset of sarcomas in these families is significantly younger than for sarcomas in the general population (47y versus 59y in Victorian Cancer Registry). Non-sarcoma cancers in these families also occur at a younger age than in the VCR (58y versus 65y, *P*<0.001). A survey of recognisable familial cancer patterns identified 56% without a family history, 23% conforming to the Eeles’ criteria for LF–like syndrome (LFL), and 1% (4 families) with classic LFS. Another 14% of families have odd patterns of familial clustering without conforming to any known syndrome. There is a 2.7-fold excess of cases of Hodgkin’s lymphoma (HL), which is not explained by radiation exposure. The age of onset of the HL cases is 31y (compared with 42y in the VCR), while the age of onset of the sarcomas in the HL families is 40y.

A survey is being conducted of the incidence of mutations in TP53 in unselected patients with sarcoma to determine a) the frequency of true LFS; b) the incidence of unsuspected de novo mutations. A combination of sequencing (exons 2-11) and multiplex ligation-dependent probe amplification (MLPA) was used to detect mutations in TP53. In addition, CHEK2 exon 9 deletions and the del1100C variant were screened. Full pedigree and pathologic information was available on all subjects. In the first 272 probands, 12 cases (4%) of pathogenic mutations in TP53 were identified. The majority of mutations affected the DNA binding domain, but one mutation affected exon 2 (frameshift) and one the tetramerization domain. We have identified only one mutation by MLPA. Four families fulfilled criteria for LFS, two met the Eeles’ criteria for LFL, and in 6 cases there was no family history. The pathogenicity of these apparently de novo cases is supported by the fact that 4 individuals without family history had two or more cancers, mostly outside a radiation field. The average age of first cancer onset in affected individuals is 40 years, or 15 years younger than the ISKS cohort. One further individual, who lacked a family history but developed two cancers, carried a separate mutation in CHEK2.

We conclude that perhaps one in twenty-five individuals with adult-onset sarcoma carries a pathogenic germline mutation in TP53 or CHEK2. Strikingly, over half of the affected do not have a family history. These findings have immediate clinical management implications regarding screening, treatment and reproductive choices in the sarcoma population. We propose to conduct a prospective study of cancer screening in families affected by inherited or de novo mutations in TP53, in order to develop an evidence-based risk modification program for affected individuals.

